# Lithopedion diagnosed during infertility workup: a case report

**DOI:** 10.1186/2193-1801-3-151

**Published:** 2014-03-19

**Authors:** Robin Medhi, Banashree Nath, Mangal Prasad Mallick

**Affiliations:** Department of obstetrics and gynaecology, Silchar Medical College and Hospital, Silchar, Assam 788014 India; Department of obstetrics and gynaecology, Ramakrishna Mission Seva Pratisthan, 99 Sarat Bose Road, Kolkata, West Bengal 700026 India

## Introduction

Lithopedion is an exceedingly rare entity in the modern era of medicine. Since the earliest case discovered in 1582 in France (Bondeson 1996), less than 300 cases of lithopedion have been reported (Irick et al. 1970; Frayer and Hibbert 1999; Spiritos et al. 1987). However in places with limited access to health care facilities and poor health awareness, lithopedion on rare occasions can baffle physicians with its appearance. Here we report a case of lithopedion in a young woman of 20 years resulting from ruptured ectopic pregnancy who attended our hospital for infertility.

## Case report

**Figure 1:**
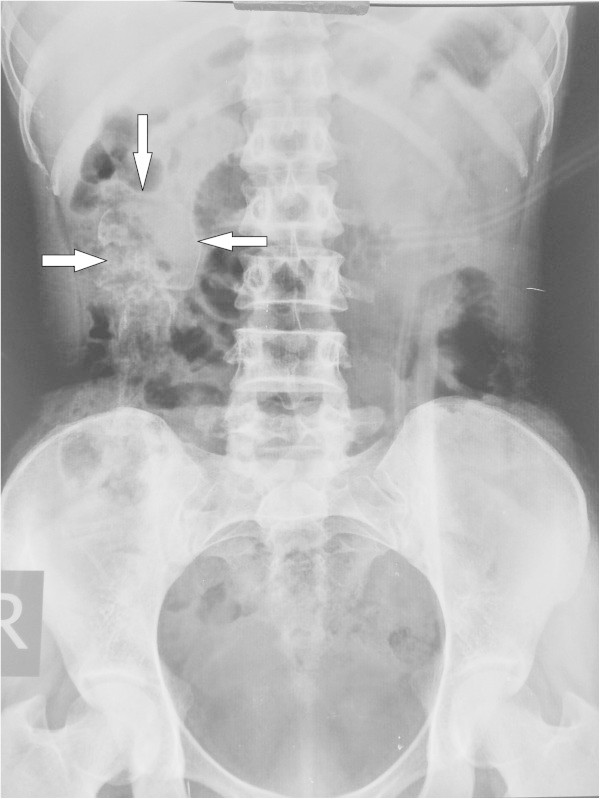
**Abdominal radiograph showing radio-opaque shadow of calcified skull of lithopedion.**

A 20 years old lady married for 2 years came with complaints of inability to conceive. Her menstrual cycles were regular except for a single missed cycle which occurred about 18 months back. She did not visit any doctor for confirmation of pregnancy. She resumed her menstruation thereafter and continued to have it till date. She however had occasional pain abdomen which was relieved by analgesics she purchased over the counter. Physical examination revealed a lump in the right lumber region hard in consistency with restricted mobility and tender on movement. Laboratory workup revealed no abnormal values. X-ray of abdomen and pelvis in erect posture revealed radio-opaque shadow resembling foetal skeleton in right lumber region (Figure 1). Ultrasound examination confirmed intraperitoneal dead, calcified foetus of approximately 17 weeks gestational age along with an echogenic mass in left adnexa.

**Figure 2:**
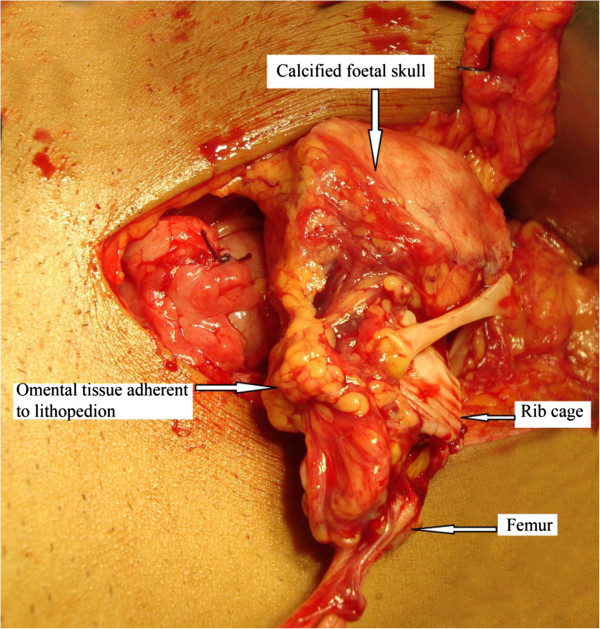
**Lithopedion in the process of extraction from abdominal cavity showing adherent omental tissue to calcified mass.**

**Figure 3:**
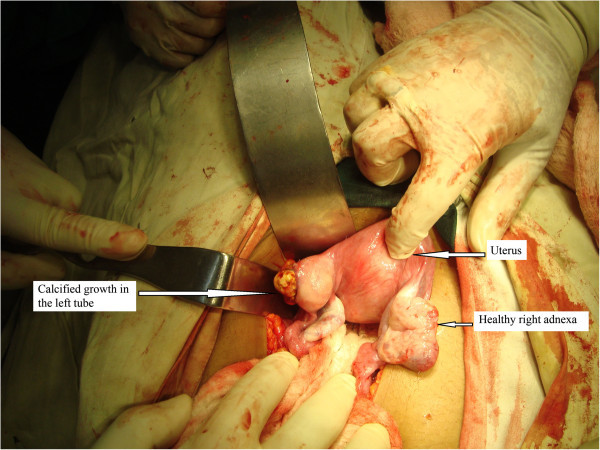
**Rent in the left fallopian tube filled with growth of calcified chorionic tissue showing the site of rupture of tubal ectopic pregnancy.**

With these findings, a provisional diagnosis of lithopedion was made and laparotomy was planned. A hard globular mass adherent to the omentum was found in the right flank. The mass was dissected off the omental tissue and a calcified foetal skeleton was recovered (Figure 2). Fallopian tube on the left side in the isthmic region contained a rent with a calcified growth that filled the tube causing a localized distension (Figure 3). This was confirmed to be calcification of degenerated chorionic tissue by histopathology with no evidence of inflammation. Left sided salphingectomy was done. Contralateral tube and bilateral ovaries were normal. Pouch of douglas was free of adhesion. Postoperative recovery was uneventful and patient was discharged on 7th postoperative day. Only 4 months after the surgical procedure, the patient again visited our OPD with complaints of cessation of menstruation for 2 months. Intrauterine gestation was confirmed. Patient attended antenatal clinic regularly. She subsequently delivered at 38 weeks a healthy female baby weighing 2.8 kg spontaneously.

## Discussion

Lithopedion is a greek word which means ‘stonechild’. This rare event occurs in 0.0054% of all gestations (Ede et al. 2011). Incidence of secondary abdominal pregnancy is 1 in 11,000 pregnancies. Lithopedion occurs in 1.5 to 1.8% of these cases (Costa et al. 1991; Frayer and Hibbert 1999).

Lithopedion describes an intraabdominal calcified dead fetus. A lithopedion can result from a primary abdominal pregnancy, or from a secondary abdominal implantation following tubal abortion or rupture of tubal or intrauterine pregnancy. It occurs when a sterile extrauterine fetus survives for more than 3 months in abdominal cavity and escapes medical discovery along with minimal and sluggish circulation inviting calcium deposition (Irick et al. 1970; Frayer and Hibbert 1999; Costa et al. 1991). Secondary abdominal implantation is one of rarest consequence of ruptured tubal pregnancy and the formation of lithopedion out of it is even rarer.

**Figure 4:**
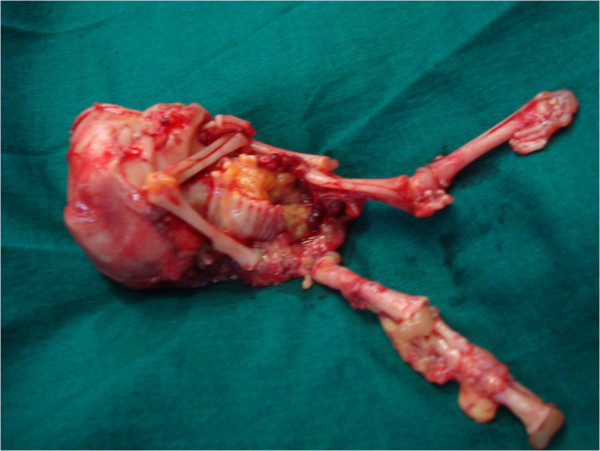
**Lithopedion after extraction.**

Age of the patients in various case reports ranged from 23 to 100 years at the time of diagnosis (Lachman et al. 2001). The occurrence of this rare condition in a woman of 20 years in our case is quite unusual. Preoperative diagnosis of lithopedion was made with simple diagnostic tools averting the need for expensive, sophisticated gadgets. This is specially rewarding in areas with scarce diagnostic facilities where from these rare cases of lithopedion are reported. The formation and diagnosis of lithopedion in our case (Figure 4) occured in less than 18 months duration since the gestational age of the recovered stonechild far exceeds an estimated period of 8 weeks when the tubal rupture is assumed to occur. This therefore is the earliest period of diagnosis in literature with various case reports citing the period of retention to be 4 to 60 years (Ede et al. 2011). The tubal rupture which resulted in secondary abdominal pregnancy is evident from the rent in the tube that was filled with calcified growth of degenerated chorionic tissue. This synchronous evidence of cause and effect is unique in itself rendering this the first of its kind. In view of the absence of salphingitis or adhesion, the obvious cause of ectopic pregnancy could not be elicited. However factors causing infertility could probably be imputed to lithopedion on the right side resulting in distorsion of pelvic anatomy hindering ovum pickup. Removal of lithopedion restored the tubo-ovarian relationship resulting in conception within 2 months of surgical intervention. Salphingectomy was adopted as the procedure of choice as the tube was grossly damaged. Surgical intervention is hence well justified in this young lady in contrast to conservative approach in view of the long survival years that can ensue her to various complications.

A rare entity though, lithopedion is not exinct and its diagnosis should not be missed in young infertile patients where period of retention may be small with minimal symptoms and vague obstetrical history. Appropriate history and keen suspicion in such cases from areas with limited access to healthcare facilities not only helps in diagnosis but can avert the dreadful complications it can accrue in course of time.

## Consent

Written informed consent was obtained from the patient for the publication of this report and any accompanying images.
